# Bacteremia associated with pressure ulcers: a prospective cohort study

**DOI:** 10.1007/s10096-018-3216-8

**Published:** 2018-02-26

**Authors:** Elena Espejo, Marta Andrés, Rosa-Maria Borrallo, Emma Padilla, Enric Garcia-Restoy, Feliu Bella, Complex Wounds Working Group

**Affiliations:** 1 Infectious Diseases Unit, Internal Medicine Service, Hospital de Terrassa (Consorci Sanitari de Terrassa), Ctra. Torrebonica s/n, 08227 Terrassa, Spain; 2 Internal Medicine Service, Hospital de Terrassa (Consorci Sanitari de Terrassa), Ctra. Torrebonica s/n, 08227 Terrassa, Spain; 3 Microbiology Laboratory, Catlab, Vial Sant Jordi s/n, Pol. Ind. Can Mitjans, 08232 Viladecavalls, Spain

**Keywords:** Pressure ulcers, Decubitus ulcers, Bacteremia, Bloodstream infection

## Abstract

The objective of this study is to evaluate the clinical and microbiological characteristics of bacteremia associated with pressure ulcers (BAPU) and factors associated with mortality. This study was a prospective observational cohort study of patients with BAPU at a teaching hospital between January 1984 and December 2015. Fifty-six episodes were included. The incidence of BAPU decreased from 2.78 cases per 10,000 hospital discharges in the period from 1984 to 1999 to 1.05 cases per 10,000 hospital discharges in the period from 2000 to 2015 (*p* < 0.001). In 20 cases (35.7%), the bacteremia was hospital-acquired, since it occurred more than 48 h after the hospital admission. The most frequent microorganisms isolated in blood culture were *Staphylococcus aureus*, *Proteus* spp., and *Bacteroides* spp. The bacteremia was polymicrobial in 14 cases (25.0%). Overall mortality was observed in 23 episodes (41.1%). The risk factors independently associated with mortality were hospital-acquired bacteremia (odds ratio [OR] 5.51, 95% confidence interval [95%CI] 1.24–24.40), polymicrobial bacteremia (OR 6.88, 95%CI 1.22–38.89), and serum albumin <23 g/L (OR 8.00, 95%CI 1.73–37.01). BAPU is an uncommon complication of pressure ulcers and is mainly caused by *S. aureus*, *Proteus* spp., and *Bacteroides* spp. In our hospital, the incidence of BAPU has declined in recent years, coinciding with the implementation of a multidisciplinary team aimed at preventing and treating chronic ulcers. Mortality rate is high, and hospital-acquired bacteremia, polymicrobial bacteremia, and serum albumin < 23 g/L are associated with increased mortality.

## Introduction

Pressure ulcers are a frequent complication in patients with limited mobility. They mainly develop in elderly patients with debilitating diseases and in patients with spinal cord injuries. A prevalence of 11–25% in nursing homes and 3–11% in hospitals is estimated [1–4]. The greater longevity of the population and the increase in spinal cord injuries due to traffic accidents has contributed to the increased incidence of pressure ulcers.

The main infectious complications that can develop from pressure ulcers are cellulitis, abscess, osteomyelitis, and bacteremia [5, 6]. Bacteremia is an infrequent complication but can lead to significant mortality [6, 7]. Published studies of bacteremia associated with pressure ulcers (BAPU) are scarce, and ulcers as the origin of the bacteremia are not always well-documented [8–10]. On the other hand, large published case series of bloodstream infections do not usually specifically identify those originating from pressure ulcers, rather these cases are usually included in the group of bacteremia originating from skin and soft tissue [11–14].

As part of our prospective study of all bloodstream infections, we have specifically studied the episodes of BAPU during a period of 32 years.

## Methods

### Setting, patients and study design

All consecutive patients with BAPU admitted between January 1984 and December 2015 in a 350-bed teaching hospital serving a population of approximately 200,000 were prospectively included in the study. If a patient presented for more than one episode of bloodstream infection caused by the same microorganism separated by less than 4 weeks, only the first episode was analyzed. Criteria for inclusion in the study included all of the following: one or more true positive blood cultures; presence of one or more pressure ulcers; positive culture of the ulcer, including at least the microorganism(s) isolated in the blood culture, with identical resistance phenotype; and exclusion of other sources of bloodstream infection. In order to rule out other sources of bacteremia, a chest x-ray and a urinary cell count were obtained in all cases, as well as a urine culture when leukocyturia existed. If there was any symptom or sign suggesting an intra-abdominal infection, ultrasound and/or abdominal CT were performed. Cases were excluded if these or other examinations suggested that another source of bacteremia was possible.

### Microbiological studies

Blood cultures were requested by the patients’ physicians. In each case two sets of blood cultures were obtained. From 1984 to 1987, a 5-ml volume of venous blood was inoculated aseptically into the broth phase of Castaneda’s biphasic medium consisting of brain heart infusion agar and broth (High Media, Mumbai, India). The media were incubated at 37 °C. From 1988 to 2015 blood cultures were collected in bottles with aerobic and anaerobic media without resins and processed with the BacT/Alert automated system (bioMérieux). Samples for ulcer culture were obtained by swabbing, perilesional puncture, or surgical debridement, depending on the characteristics of the ulcers. All samples were seeded in aerobiosis and anaerobiosis on blood agar, chocolate agar, MacConkey agar, anaerobic selective medium and thioglycolate liquid medium (bioMérieux). Identification of microorganisms was performed using biochemical tests included in the commercial systems Api (bioMérieux) and Microscan (Beckman Coulter). The minimum inhibitory concentration (MIC) was determined by microdilution (Microscan automated system, Beckman Coulter) for aerobic microorganisms, and using the disc-diffusion or the E-test method for anaerobic bacteria. The results were interpreted according to the criteria of the Clinical Laboratory Standards Institute (CLSI; formerly NCCLS) until 2013 and according to the criteria of the European Committee on Antimicrobial Susceptibility Testing (EUCAST) from 2014 on.

### Definitions

All cases were directly evaluated at admission by one of the authors (EE, MA, RMB, or FB). The data for each episode were recorded on a predefined form with 97 items and stored in a database.

Pressure ulcers were defined as localized lesions of the skin and underlying tissue caused by compression between bony prominences and external surfaces. Ulcers attributable to vasculopathy, peripheral neuropathy, surgery, or trauma were excluded. Ulcers were classified according to the National Pressure Ulcer Advisory Panel criteria [15]. When more than one ulcer was present in a patient, samples were taken for culture of all ulcers with local signs of infection, and the stage of the most advanced ulcer was awarded to the case.

Bacteremia was considered to be hospital-acquired, healthcare-related or community-acquired according to Friedman criteria [16]. Information about underlying medical conditions was obtained by reviewing patients’ medical records. Patients were considered as having diabetes mellitus if they required insulin therapy or oral hypoglycaemic agents before the episode of bacteremia. Cognitive impairment was defined as stage 5–7 of the Reisberg scale [17]. Patients were classified into three categories on the McCabe scale [18], according to their prognosis before the onset of bacteremia: non-fatal, if death was expected within a period > 5 years; ultimately fatal, if death was expected between 1 and 5 years; and rapidly fatal, if death was expected within the following year. Shock was defined as a systolic blood pressure < 90 mmHg that was unresponsive to fluid administration or required vasoactive drugs.

Initial empirical antibiotic treatment was considered appropriate when at least one in vitro active antibiotic was included in the treatment within the first 24 h of the onset of bacteremia.

Overall mortality rate was defined as death by any cause within the first 30 days since the onset of bacteremia. Death was considered directly related to bacteremia when it occurred within 7 days after the last positive blood culture or, if later, when there were signs of uncontrolled infection or complications of bacteremia.

### Statistical analysis

Incidence of BAPU was calculated as episodes of bloodstream infection associated with pressure ulcers per 10,000 hospital discharges.

Descriptive analysis was performed using means with standard deviation (SD) for continuous variables and percentages for categorical variables. Continuous variables were compared using the Mann-Whitney *U* test. Categorical and stratified continuous variables were compared using the chi-square test or the Fisher’s exact test as appropriate. Odds ratios (ORs) and 95% confidence intervals (CI) were calculated. Statistical significance was defined as *p* < 0.05. Multivariate logistic regression analysis of factors potentially associated with mortality was performed including all the variables that achieved *p* < 0.20 in the univariate analysis. The SPSS (version 15.0) software package was used for the statistical analysis.

## Results

During the study period, 56 consecutive episodes of BAPU were identified in 53 patients. The incidence of BAPU was 1.70 episodes per 10,000 adult patient discharges, over the whole study period. This incidence was significantly higher in the 1984–1999 period (2.78 episodes per 10,000 discharges) compared to the 2000–2015 period (1.05 episodes per 10,000 discharges; *p* < 0.001).

The bacteremia was hospital-acquired in 20 cases (35.7%), healthcare-related in 15 cases (26.8%), and community-acquired in 21 cases (37.5%). The baseline and clinical characteristics are summarized in Table 1.

**Table 1:** Baseline and clinical characteristics of 56 episodes of bacteremia associated with pressure ulcers

Characteristic	*n* (%)
Age (years; mean, SD)	75.9 (14.1)
Gender	
Male	27 (48.2)
Female	29 (51.8)
Underlying medical conditions	
Cognitive impairment	29 (51.8)
Diabetes mellitus	22 (39.3)
Chronic renal failure	11 (19.6)
Tetraplegy/paraplegy	9 (16.1)
Cerebrovascular disease	7 (12.5)
Hip fracture	5 (8.9)
Malignancy	5 (8.9)
Chronic obstructive pulmonary disease	3 (5.4)
Parkinson disease	2 (3.6)
Multiple sclerosis	1 (1.8)
HIV infection	1 (1.8)
McCabe scale	
Non-fatal	41 (73.2)
Ultimately fatal	13 (23.2)
Rapidly fatal	2 (3.6)
Clinical characteristics and laboratory findings	
Axillary temperature > 37.5 °C	44 (78.6)
Shock	8 (14.3)
Disseminated intravascular coagulation	3 (5.4)
Leukocyte count (no./μL; mean, SD)	14,412 (6530)
Hemoglobin (g/dL; mean, SD)	10.6 (2.3)
Serum albumin (g/L; mean, SD)	23.9 (4.6)

Microorganisms isolated from blood culture and ulcer culture are detailed in Table 2. Bacteremia was polymicrobial in 14 cases (25.0%) while the ulcer culture was polymicrobial in 41 cases (73.2%). The most frequent causative agent of BAPU was *Staphylococcus aureus* followed by *Proteus* spp., *Bacteroides* spp., and *Escherichia coli.* Only 4 episodes of BAPU were caused by methicillin-resistant *S. aureus* (MRSA), all from 2002, and only one episode was caused by ESBL-producing *Enterobacteriaceae*. No bacterial isolates were carbapenem-resistant. Of the four episodes caused by *Pseudomonas* spp., two were hospital-acquired and two were community-acquired. The most frequently isolated microorganisms from the ulcer cultures were *E. coli* and *Proteus* spp., followed by *S. aureus*, *Bacteroides* spp., *Pseudomonas* spp., and *Enterococcus* spp.

**Table 2:** Microbiology in 56 episodes of bacteremia associated with pressure ulcers

Microorganism^a^	Blood culture^b^ *n* (%)*	Ulcers culture^c^ *n* (%)*
*Staphylococcus aureus*	17^d^ (30.4)	20^e^ (35.7)
*Proteus* spp.	16 (28.6)	26^f^ (46.4)
*Bacteroides* spp.	13 (23.2)	17 (30.4)
*Escherichia coli*	8 (14.3)	27 (48.2)
*Pseudomonas* spp.	4 (7.1)	13 (23.2)
Anaerobic gram-positive cocci	4 (7.1)	5 (8.9)
*Streptococcus viridans*	3 (5.4)	5 (8.9)
Coagulase-negative staphylococci	3 (5.4)	3 (5.4)
*Enterococcus* spp.	2 (3.6)	11 (19.6)
*Streptococcus agalactiae*	1 (1.8)	2 (3.6)
*Candida* spp.	1 (1.8)	4 (7.1)
*Klebsiella pneumoniae*	–	1 (1.8)
*Citrobacter freundii*	–	1 (1.8)

*Percentages are calculated among the total of isolates

^a^Samples for ulcers culture were obtained from swabbing in 23 cases, from deep tissue at the debridement in 30 cases, and from perilesional puncture in 3 cases

^b^The bacteremia was polymicrobial in 14 cases (25.0%)

^c^The ulcer culture was polymicrobial in 41 cases (73.2%)

^d^Methicillin-resistant *S. aureus* (MRSA) in 4 cases

^e^Methicillin-resistant *S. aureus* (MRSA) in 8 cases

^f^Extended spectrum β-lactamase (ESBL)-producing *Proteus mirabilis* in one case

In 49 cases (87.5%), the ulcers responsible for bacteremia were already present at admission, whereas in 7 cases (12.5%) they were acquired during the hospital stay. The percentage of hospital-acquired ulcers during the period from 1984 to 1999 (17.6%) was higher than in the period from 2000 to 2015 (4.5%), although the difference was not statistically significant (*p* = 0.22). In 42 cases (75.0%) there was more than one ulcer. The characteristics of the ulcers are shown in Table 3.

**Table 3:** Ulcer characteristics in 56 episodes of bacteremia associated with pressure ulcers

Characteristic	*n* (%)
Ulcers location^a^	
Sacrum	45 (80.4)
Heels	23 (41.1)
Trochanter area	17 (30.4)
Ischial area	9 (16.1)
Trunk	6 (10.7)
Buttocks	6 (10.7)
Malleolus	5 (8.9)
Lower legs	4 (7.1)
Lumbar area	2 (3.6)
Other	3 (5.4)
Ulcers staging	
Stage II	3 (5.4)
Stage III	12 (21.4)
Stage IV	41 (73,2)
Signs of local infection	
Purulent exudate	35 (62.5)
Surrounding inflammation	34 (60.7)
Friable granulation tissue	40 (71.4)
Foul odor	24 (42.9)
Underlying abscess	17 (30.4)
Osteomyelitis beneath the ulcer	13 (23.2)

^a^There was more than one ulcer in 42 cases (77.0%)

Empirical antibiotic treatment was appropriate in 44 cases (78.6%). All patients were treated locally with ulcer cleaning. Surgical debridement was performed in 36 cases (64.3%). Overall mortality was observed in 23 cases (41.1%). Death was considered directly related to bacteremia in 12 cases (21.4%). The age of the patients who died (77.5 ± 14.5 years) was not significantly different from the age of the patients who survived (74.7 ± 13.8 years; *p* = 0.46). Patients who died had significantly lower serum albumin (21.8 ± 4.9 g/L) than patients who survived (25.3 ± 4.0 g/L; *p* = 0.0067). Table 4 summarizes the risk factors for overall mortality rate. Variables associated with overall mortality in the univariate analysis were hospital-acquired bacteremia, polymicrobial bacteremia, failure to perform surgical debridement of the ulcer, and serum albumin below 23 g/L. Independent risk factors for mortality were hospital-acquired bacteremia, polymicrobial bacteremia, and serum albumin below 23 g/L.

**Table 4:** Risk factors for overall mortality rate by univariate and multivariate analysis

Characteristics*	Survived	Died	*p* value	Adjusted OR (95%CI)	*p* value
	*n* = 33 (%)	*n* = 23 (%)			
Age > 80 years	12 (36.4)	11 (47.8)	0.391		
Rapidly or ultimately fatal underlying disease	7 (21.2)	8 (34.8)	0.259		
Hospital-acquired bacteremia	8 (24.2)	12 (52.2)	0.032	5.51 (1.24–24.40)	0.024
Polymicrobial bacteremia	5 (15.2)	9 (39.1)	0.041	6.88 (1.22–38.89)	0.028
Serum albumin < 23 g/L	9 (27.3)	14 (60.9)	0.011	8.00 (1.73–37.01)	0.007
Shock	2 (6.1)	6 (26.1)	0.053	4.68 (0.61–35.54)	0.135
No surgical debridement	8 (24.2)	12 (52.2)	0.032	3.22 (0.77–13.45)	0.108
Axillary temperature > 37.5 °C	6 (18.2)	6 (26.1)	0.522		
Inappropriate antibiotic treatment	8 (24.2)	4 (17.4)	0.743		

*The variables that achieved a *p* < 0.20 in the univariate analysis were included in the multivariate analysis

## Discussion

When a patient with decubitus ulcers presents with fever in the absence of other foci of infection, bacteremia due to the ulcers should be suspected. On the other hand, the appearance of fever immediately after debridement of an ulcer may reflect the existence of transient bacteremia related to the debridement [10]. Although bacteremia is a well-known complication of pressure ulcers, its incidence is not well established and studies on this subject are scarce [8–10]. The present study constitutes the largest case series of BAPU published so far. It has been prospectively obtained in a single institution, over 32 years, applying strict diagnostic criteria. In the whole study period, 1.7 episodes of BAPU per 10,000 hospital discharges were observed, which coincides with the incidence observed by Bryan [9]. It is noteworthy that in the period from 2000 to 2015, the incidence was significantly lower than in the period from 1984 to 1999 (1.05 vs. 2.78 episodes per 10,000 discharges). This reduction coincided with the implementation of a multidisciplinary team aimed at preventing and treating chronic ulcers, both in the hospital and in primary care centers dependent on our institution. Although only 12.5% of the ulcers were acquired in the hospital, 35.7% of the episodes of bacteremia were hospital-acquired. Hospital admission for an intercurrent illness is likely to be a risk factor for developing bacteremia from pre-existing ulcers, either due to further deterioration of the host defense mechanisms or changes in the microorganisms that colonize the ulcer.

A striking finding in this study was the high frequency of polymicrobial bacteremia, already observed in previous studies [8, 9, 19], as a consequence of the typical polymicrobial colonization of ulcers. *S. aureus*, which was the most frequently isolated microorganism, has a special ability to spread to the bloodstream from pressure ulcers. Thus, in 17 of the 20 cases in which *S. aureus* was recovered from the ulcers, this was the same microorganism that was responsible for the bacteremia. The same is not true of *Enterococcus* spp., which was recovered in 11 cases in ulcer culture but in only 2 cases was it responsible for the bacteremia. Among *Enterobacteriaceae*, *Proteus* spp. originating from pressure ulcers have a special ability to cause bacteremia and were responsible for more than a quarter of the episodes in this study. The prevalence of *Proteus* spp. has also been observed in other studies [8, 9], although a satisfactory explanation for this predominance has not yet been formulated. By contrast, while *E. coli* was recovered in the culture of ulcers in almost half of the cases, only in 8 cases was *E. coli* responsible for the bacteremia. Several researchers have highlighted the importance of anaerobic microorganisms, finding that the devitalized ulcer tissue provides an adequate environment for persistence and proliferation of such organisms [9, 20, 21]. It is worth noting the presence of *Bacteroides fragilis*, which reflects fecal contamination of ulcers, especially those in the sacral location [22]. This pathogen was responsible for 23% of the episodes of BAPU in this study.

We did not identify any clinical or epidemiological factors that allowed reliable prediction of blood culture findings. In addition, the history of a previous episode of BAPU does not appear to be useful in predicting the causative pathogen, as demonstrated in three patients in our study who presented for recurrent bacteremia due to a pathogen other than the one that caused the initial episode. This is because the local infection of the ulcers is usually polymicrobial and, likewise, the chances of colonization by new microorganisms are high. Therefore, when an episode of BAPU is suspected, empiric antibiotic treatment should adequately cover *S. aureus*, gram-negative enteric bacilli, and anaerobic microorganisms, including *B. fragilis*, taking into account the local resistance rates. Once the blood culture results become available, antibiotic treatment should be appropriately adjusted and directed to the microorganisms responsible for the bacteremia.

Pressure ulcers may constitute an important reservoir of multiresistant microorganisms, especially MRSA, with the consequent risk of bacteremia by this pathogen [23–26]. It has been shown that the presence of a chronic wound is an independent risk factor for the persistence of MRSA colonization after hospital discharge [27]. In this study, only four episodes of BAPU were caused by MRSA. This may be due in part to the active search-and-destroy policy against MRSA adopted at our hospital where annual rates of methicillin resistance in *S. aureus* isolated from clinical samples in the last 10 years have ranged from 4.5 to 13.8% (unpublished data), lower than the percentages observed in most hospitals in our country [28].

Osteomyelitis is a well-known complication of pressure ulcers. It should be suspected in ulcers with a torpid course that do not improve despite intensive local treatment [29, 30]. A positive culture of a bone biopsy obtained through the ulcer may reflect only the colonization of the adjacent soft tissue, and the gold standard for the diagnosis of osteomyelitis is pathological examination of bone tissue [31]. Among non-invasive diagnostic methods, magnetic resonance imaging (MRI) offers the highest sensitivity and specificity [32]. A limitation of this study is that the presence of osteomyelitis was not systematically investigated, leaving the practice of MRI and bone biopsy to the discretion of the primary clinician. Therefore, it is possible that this complication has been underdiagnosed.

Overall mortality was high, although slightly lower than previously reported [8, 9]. In only half of the cases, death was directly related to bacteremia, a percentage similar to that previously described [9]. Hospital-acquired bacteremia, polymicrobial bacteremia, and serum albumin of less than 23 g/L were independent risk factors for 30-day mortality rate. In addition to being a marker of protein malnutrition, hypoalbuminemia may appear in a wide constellation of clinical situations, as a consequence of reduced hepatic synthesis, renal or intestinal losses, or transcapillary escape to the interstitial space [33, 34]. Albumin has been considered a negative acute-phase protein, since its serum concentration is prone to decrease in trauma and sepsis, and hypoalbuminaemia has proven to be a marker of poor prognosis in different clinical settings, including bacteremia [35, 36].

Surgical debridement of the ulcer is an important element in the management of BAPU. It is a safe procedure in patients with advanced ulcers, despite the comorbidities that these patients usually present [37]. Galpin et al. [8] observed cases of persistent bacteremia that were only resolved after surgical ulcer debridement, as well as a lower mortality among patients undergoing surgical debridement, as occurred in this study.

Finally, although broad-spectrum antibiotic treatment and surgical debridement are important elements for the management of BAPU, the most effective measure is the prevention of pressure ulcers through early detection of patients at risk and the adoption of appropriate preventive measures [38–40].

In conclusion, BAPU is an uncommon complication of pressure ulcers and is mainly caused by *S. aureus*, *Proteus* spp., and *Bacteroides* spp. Its incidence in our institution has declined in recent years. Mortality rate is high and hospital-acquired bacteremia, polymicrobial bacteremia, and serum albumin < 23 g/L are associated with increased mortality.
